# Cataract in the Horse

**Published:** 1896-09

**Authors:** 


VETERINARIAN CAPTAIN F. SMITH, in the June number of the *Journal of Comparative Pathology and Therapeutics*, in an excellent article on "Cataract in the Horse," brings out the following prominent points:

1. The great value of the ophthalmoscope in the determination of cataract.

2. The necessity for examining the lens when the pupil is moderately dilated.

3. The concealment of the cataracts by the iris and corpora nigra.

4. The fact that a dot of opacity, even at the centre of the lens, may be compatible with normal acuteness of vision.

5. That partial opacity in the form of dots is more common than general opacity, and that such dots, if hereditary, as they probable are, show no disposition to spread or invade the entire substance of the lens.

6. Though cataract constitutes unsoundness, it need not be a cause of rejection, but a purchaser should have the acuteness of vision guaranteed.

